# Thromboelastometry for the assessment of coagulation abnormalities in early and established adult sepsis: a prospective cohort study

**DOI:** 10.1186/cc7765

**Published:** 2009-03-30

**Authors:** Fritz Daudel, Ulf Kessler, Hélène Folly, Jasmin S Lienert, Jukka Takala, Stephan M Jakob

**Affiliations:** 1 Department of Intensive Care Medicine, Inselspital, Bern University Hospital and University of Bern, Freiburgstrasse, 3010 Bern, Switzerland; 2 Department of Pediatric Surgery, Inselspital, Bern University Hospital and University of Bern, Freiburgstrasse, 3010 Bern, Switzerland

## Abstract

**Introduction:**

The inflammatory response to an invading pathogen in sepsis leads to complex alterations in hemostasis by dysregulation of procoagulant and anticoagulant factors. Recent treatment options to correct these abnormalities in patients with sepsis and organ dysfunction have yielded conflicting results. Using thromboelastometry (ROTEM^®^), we assessed the course of hemostatic alterations in patients with sepsis and related these alterations to the severity of organ dysfunction.

**Methods:**

This prospective cohort study included 30 consecutive critically ill patients with sepsis admitted to a 30-bed multidisciplinary intensive care unit (ICU). Hemostasis was analyzed with routine clotting tests as well as thromboelastometry every 12 hours for the first 48 hours, and at discharge from the ICU. Organ dysfunction was quantified using the Sequential Organ Failure Assessment (SOFA) score.

**Results:**

Simplified Acute Physiology Score II and SOFA scores at ICU admission were 52 ± 15 and 9 ± 4, respectively. During the ICU stay the clotting time decreased from 65 ± 8 seconds to 57 ± 5 seconds (*P *= 0.021) and clot formation time (CFT) from 97 ± 63 seconds to 63 ± 31 seconds (*P *= 0.017), whereas maximal clot firmness (MCF) increased from 62 ± 11 mm to 67 ± 9 mm (*P *= 0.035). Classification by SOFA score revealed that CFT was slower (*P *= 0.017) and MCF weaker (*P *= 0.005) in patients with more severe organ failure (SOFA ≥ 10, CFT 125 ± 76 seconds, and MCF 57 ± 11 mm) as compared with patients who had lower SOFA scores (SOFA <10, CFT 69 ± 27, and MCF 68 ± 8). Along with increasing coagulation factor activity, the initially increased International Normalized Ratio (INR) and prolonged activated partial thromboplastin time (aPTT) corrected over time.

**Conclusions:**

Key variables of ROTEM^® ^remained within the reference ranges during the phase of critical illness in this cohort of patients with severe sepsis and septic shock without bleeding complications. Improved organ dysfunction upon discharge from the ICU was associated with shortened coagulation time, accelerated clot formation, and increased firmness of the formed blood clot when compared with values on admission. With increased severity of illness, changes of ROTEM^® ^variables were more pronounced.

## Introduction

Organ failure contributes cumulatively to mortality in patients with sepsis [1]. One of the mechanisms that is believed to contribute to the pathogenesis of organ failure in sepsis is microvascular thrombosis [2-5]. Pathways involved in the prothrombotic state of critically ill patients include tissue factor-mediated thrombin generation and impaired anticoagulant and fibrinolytic mechanisms [6]. Continuing consumption of platelets and coagulation factors may cause overt disseminated intravascular coagulation and carry risk for bleeding diathesis [7]. In severe inflammation, coagulation-regulating systems appear to be defective, primarily as a result of endothelial dysfunction [8,9]. However, the effect of anticoagulant therapies on outcome is controversial [10-13].

In clinical practice, routinely performed blood coagulation tests only incompletely mirror sepsis-induced coagulation abnormalities, and hypercoagulation in particular is not detected. More advanced laboratory analyses or experimental methods to monitor coagulation in critically ill patients, including tissue factor levels [14], prothrombin fragments F_1 _and F_2 _[15], thrombin-antithrombin (TAT) [16,17], and thrombomodulin expression [18], have not yet been introduced into routine clinical management.

End-points of routine coagulation tests occur early in the hemostatic process, whereas thromboelastography measures the viscoelastic characteristics of blood clot formation in a whole blood assay, and it may therefore provide additional information on coagulation. Thromboelastography assesses the influence of plasmatic factors and platelets during all phases of the coagulation process. Thus, it permits one to evaluate the initiation of coagulation, the propagation of clot formation, and the final firmness of the blood clot.

Thromboelastography has gained importance in the management of bleeding disorders in trauma and surgical patients [19-21]. It has also been used to evaluate alterations in hemostasis in *in vitro *models of endotoxemia or at a single time point in patients with sepsis [22-25]. However, the evolution of disorders of hemostasis measured by thromboelastography during severe sepsis has not been investigated.

The aim of this study was to evaluate the evolution of coagulation abnormalities using thromboelastometry (ROTEM^® ^thromboelastometry; Pentapharm, Munich, Germany) in parallel with routine coagulation tests during the early phase of severe sepsis and septic shock, and to relate these abnormalities to organ dysfunction. We hypothesized that changes in ROTEM^® ^variables may be related to the evolution of organ dysfunction. ROTEM^® ^is a point-of-care device with limited susceptibility to shock and vibrations.

## Materials and methods

The study protocol for the present prospective cohort study was approved by the regional governmental ethics committee (Ethik Kommission des Kantons Bern). Written informed consent was obtained from the patients or their relatives. Thirty patients admitted to a 30-bed multidisciplinary intensive care unit (ICU) with a diagnosis of sepsis (as defined by the recommendations of the Society of Critical Care Medicine/European Society of Intensive Care Medicine/American College of Chest Physicians/American Thoracic Society/Surgical Infection Society International Sepsis Definitions Conference [26]) were enrolled in the study. Exclusion criteria were age under 18 years, pre-existent hematological disorders, current oral anticoagulants, or therapy to inhibit platelet aggregation.

### General treatment

All patients with sepsis were monitored with a radial arterial line and a central venous line, and with a pulmonary artery catheter if they were unresponsive to initial volume loading. Treatment protocols were used for hemodynamic management, weaning from mechanical ventilation, analgesia and sedation, and insulin therapy. Thromboprophylaxis in all patients was achieved with graduated compression stockings or intermittent pneumatic compression devices in addition to low-dose unfractionated heparin 10,000 units/day, in accordance with the guidelines of the American College of Chest Physicians for critically ill patients [27]. The heparin was administered as a continuous intravenous infusion.

Volume resuscitation with colloids in our patients was achieved with 6% hydroxyethyl starch 130/0.4 (Voluven^®^; Fresenius Kabi, Stans, Switzerland). Patients in the low Sequential Organ Failure Assessment (SOFA) group were administered a mean of 1,373 ± 1,929 ml over the whole study period; those in the high SOFA group received 2,523 ± 1,914 ml.

### Data acquisition

Demographic data, source of infection, and length of stay in the ICU were recorded. The Simplified Acute Physiology Score II was determined on admission. The SOFA score was assessed daily for the first 3 days after inclusion in the study and on ICU discharge. The SOFA score was developed to quantify the severity of illness based on the degree of organ dysfunction [28].

### Blood sampling

Blood samples were taken from a radial arterial line after 10 ml blood had been discarded. Blood for thromboelastometry was drawn into a 5 ml syringe and immediately anticoagulated with 0.5 ml trisodium citrate 0.106 mol/l (Sarstedt, Nümbrecht, Germany).

Routine coagulation screening, including platelet count, International Normalized Ratio (INR), activated partial thromboplastin time (aPTT), levels of coagulation factors II, V, VII and X, and fibrinogen, were measured daily for the first 3 days and upon discharge. Routine coagulation laboratory measurements and analysis of coagulation factor activity were performed using the BCS^® ^Analyzer and corresponding reagents (Siemens Health Care Diagnostics, Düdingen, Switzerland).

### Thromboelastometry

Thromboelastometry was performed using a ROTEM^® ^analyzer (Pentapharm GmbH, Munich, Germany) every 12 hours for 48 hours and at ICU discharge. The method, technique and variables of thromboelastometry were described previously [29]. Briefly, ROTEM^® ^measures viscoelastic properties of clot formation and fibrinolysis. Because of the use of a ball-bearing system for power transduction, it is less susceptible to movement and vibration.

Tests were performed using ROTEM cups and pins. The ROTEM^® ^device was tested regularly for correct function using quality control serum (ROTROL^®^; Pentapharm GmbH).

We conducted intrinsically and extrinsically activated tests (INTEM and EXTEM), in accordance with the manufacturer's recommendations (INTEM test: 20 μl CaCL_2 _0.2 mol/l, 20 μl thromboplastin-phospholipid, 300 μl blood; EXTEM test: 20 μl CaCL_2 _0.2 mol/l, 20 μl tissue factor, 300 μl blood). In order to assess a possible effect of low-dose heparin administration to our patients, we also analyzed the effects of addition of heparinase to the blood samples (HEPTEM test) and compared the results with those of the INTEM test.

The influence of thrombocytes on clot firmness was estimated with a platelet-inactivating test (FIBTEM test: 20 μl CaCL_2 _0.2 mol/l, cytochalasin D, 20 μl tissue factor, 300 μl blood). Chemicals and reagents were purchased from Pentapharm GmbH.

The variables that were measured using ROTEM^® ^thromboelastometry are the following. The clotting time (CT), the equivalent to the reaction time (r time) of conventional TEG^® ^(Haemoscope, Skokie, IL, USA), represents the initiation of coagulation. The propagation of clot formation, reflecting thrombin generation and early fibrin polymerization, is characterized by clot formation time (CFT), comparable to the clotting time (k time) and alpha angle of conventional TEG^®^. CFT is thereby defined as the time necessary to attain a clot firmness of 20 mm. The maximal clot firmness (MCF), corresponding to the maximal amplitude of conventional TEG^®^, which describes the final strength of the clot, is influenced by the fibrinogen concentration and the platelet count.

### Statistical analysis

SigmaStat version 3.5 (Systat Software, Inc., Chicago, IL, USA) was used for statistical analysis. After testing for normal distribution (Kolmogorov-Smirnov test), data were analyzed using analysis of variance for repeated measurements and the Student-Newman-Keuls test for *post hoc *comparisons or, where appropriate, Friedman analysis and the Dunn test. In order to compare coagulation profiles in groups of patients with different organ failure severity, patients were divided into two groups using median SOFA score. Differences between the two groups were analyzed using analysis of variance for repeated measurements with one grouping factor (high versus low SOFA score) and one within-subject factor (time). The correlation between routine coagulation and ROTEM^® ^variables with the severity of organ dysfunction defined by the SOFA score was studied using the nonparametric Spearman correlation (*r*) with pooled data from the continuous time points. Comparison of the clotting times between the INTEM and HEPTEM assays was analyzed with a two-sided *t*-test. For all statistical tests, significance was assumed at *P *< 0.05. Values are expressed as mean ± standard deviation or median (interquartile range) where appropriate.

## Results

### Patients

The characteristics of the included patients are shown in Table 1. Thirteen of the patients in the study suffered from severe sepsis, and 17 patients from septic shock with hypotension not reversed by volume resuscitation [26]. Median SOFA score at ICU admission was 10. There were no thrombosis and bleeding diathesis reported during the study period.

**Table 1:** Characteristics of the study population

Parameter/variable	Value
*n*	30
Age (years)	65 ± 13
Male sex (*n*)	19
Source of infection (*n*)	
Lungs	9
Catheter related	3
Endocarditis	1
Abdominal	6
Soft tissue/bone	6
CNS	3
Unknown/other	2
SAPS II score	52.3 ± 14.6
APACHE II score	29.0 ± 7.7
SOFA score	
Day 1	9.1 ± 4.0
Day 2	8.3 ± 3.8
Day 3	8.3 ± 4.2
Discharge	6.5 ± 3.7
Intensive care unit	
Length of stay (days)	6 (3/11)^a^
Mortality (n [%])	6 (20)
28 day survival (n [%])	22 (73)

^a^Length of stay is presented as median (25%/75% percentiles). All other values are presented as number or as mean ± standard deviation.

### Routine laboratory results

Coagulation factor levels, routine coagulation tests, hemoglobin, platelets, and white blood cell count are shown in Table 2. The means of aPTT and INR on admission were increased beyond the normal range (Table 2). Relevant factor activities were initially at the low end of the normal range or below it, and increased during the ICU stay. Only factor V remained close to the midpoint of reference values. The platelet counts remained within the normal range, but also increased over time. Fibrinogen levels and white blood count were raised above normal during the entire stay in the critical care unit. Spearman rank correlation between disease severity assessed by the SOFA score and the routine coagulation tests INR and aPTT revealed rather low coefficients (*r *= 0.39 and 0.51, respectively; *P *< 0.001 each).

**Table 2:** Routine coagulation tests, clotting factor levels, hemoglobin, hematocrit, white blood count and CRP

Parameter	Normal range	Day 1	Day 2	Day 3	Discharge
Platelets (g/l)	140 to 380	191 ± 117	198 ± 135	196 ± 135	364 ± 201*
INR	0.9 to 1.15	1.18 ± 0.16	1.16 ± 0.18	1.12 ± 0.15	1.09 ± 0.14
aPTT (seconds)	25 to 36	48.1 ± 11.4	48.8 ± 13.0	44.7 ± 11.2	39.8 ± 11.6*
Fibrinogen (g/l)	1.3 to 3.6	6.2 ± 2.7	5.8 ± 1.6	5.5 ± 1.6	6.0 ± 4.4
Factor II (%)	81 to 134	61 ± 22	64 ± 25	69 ± 23	76 ± 28*
Factor V (%)	78 to 153	103 ± 45	110 ± 45	114 ± 43*	116 ± 33*
Factor VII (%)	70 to 139	66 ± 29	71 ± 29	88 ± 30*	79 ± 29
Factor X (%)	68 to 145	79 ± 29	81 ± 30	90 ± 22	84 ± 21
Hemoglobin (g/l)	121 to 154	100.4 ± 14.3	98.8 ± 14.7	98.9 ± 11.6	102.2 ± 12.2
Hematocrit (%)	0.36 to 0.44	29.1 ± 4.4	29.1 ± 4.8	29.2 ± 3.8	30.4 ± 3.7
WBC (g/l)	3.5 to 10.5	16.1 ± 7.5	17.4 ± 7.3	17.0 ± 6.4	16.6 ± 6.9
CRP (mg/l)	<5	201 ± 87	202 ± 83	176 ± 94	113 ± 76*
Creatinine (μmol/l)	59 to 104	172 ± 120	159 ± 113	171 ± 115	151 ± 126
Bilirubin (μmol/l)	3 to 26	33.3 ± 34.3	37.0 ± 40.5	40.0 ± 46.2	32.8 ± 43.9

Values are expressed as Mean ± SD. *P < 0.05, versus baseline. aPTT, activated partial thromboplastin time; CRP, C-reactive protein; INR, International Normalized Ratio; WBC, white blood count.

### Thromboelastometry

Thromboelastometric variables in the entire study cohort are presented in Table 3. Average CT, CFT, and MCF remained within the normal reference values established in a multicenter study [30] (Table 3). However, during the time course of critical illness we noted decreases in CT and CFT and an increase in MCF in the tissue factor activated tests (EXTEM) compared with admission values (Table 3).

**Table 3:** Results of thromboelastometry

Parameter	Normal ranges	0 hours	12 hours	24 hours	36 hours	48 hours	Discharge	Spearman	
								
								r	*P*
CT-EXTEM	42 to 74	64.9 ± 7.6	66.0 ± 10.6	61.7 ± 9.6	63.7 ± 7.2	61.9 ± 7.3	57.4 ± 4.6*	0.09	0.36
CT-INTEM	137 to 246	195.9 ± 25.8	201.0 ± 31.4	199.8 ± 36.7	213.2 ± 41.1	216.3 ± 43.8	206.7 ± 40.6		
CT-HEPTEM	137 to 246	197.1 ± 39.3	217.2 ± 58.1	212.2 ± 47.7	212.4 ± 40.0	205.8 ± 29.6	192.8 ± 34.2		
CFT-EXTEM	46 to 148	97.0 ± 62.6	103.6 ± 79.2	99.3 ± 61.0	100.4 ± 64.8	94.5 ± 54.9	62.6 ± 30.7*	0.64	0.001
Alpha-EXTEM	63 to 81	73.9 ± 7.8	74.3 ± 7.1	74.0 ± 6.9	73.7 ± 6.8	74.5 ± 5.9	78.3 ± 4.1	-0.58	0.001
MCF-EXTEM	49 to 71	62.1 ± 11.2	62.1 ± 12.6	62.1 ± 12.1	62.1 ± 12.4	62.7 ± 11.6	67.4 ± 9.0*	-0.63	0.001
MCF-INTEM	52 to 72	63.7 ± 8.0	63.2 ± 7.6	61.9 ± 8.3	64.1 ± 7.7	64.6 ± 8.1	66.9 ± 8.4		
MCF-HEPTEM	52 to 72	61.0 ± 8.9	59.6 ± 8.0	59.1 ± 9.4	60.6 ± 9.2	61.5 ± 8.4	64.1 ± 7.8*		

Spearman rank correlation (*r*) is between the continuous values of Sequential Organ Failure Assessment (SOFA) score with thromboelastometric variables. *P < 0.05, versus baseline. alpha angle (°); CFT, clot formation time (seconds); CT, clotting time (seconds); EXTEM, activation of coagulation with tissue factor; HEPTEM, inhibition of heparin with heparinase; INTEM, activation of coagulation with thromboplastin; MCF, maximal clot firmness (mm).

**Figure 1:**
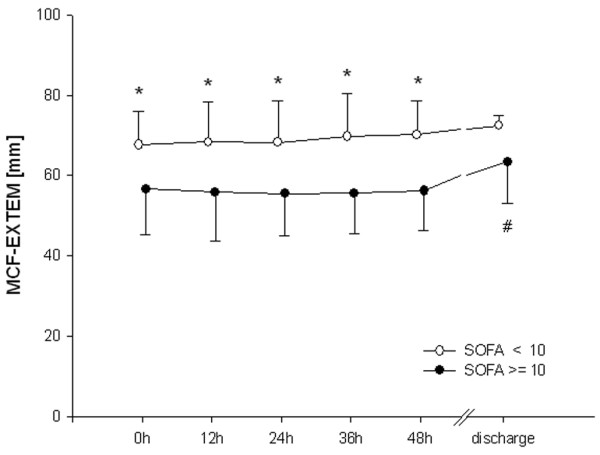
Maximal clot firmness. *P < 0.05, differences between groups; ^#^*P *< 0.05, difference from baseline value in the high Sequential Organ Failure Assessment (SOFA; ≥ 10) group. EXTEM, activation of coagulation with tissue factor; MCF, maximal clot firmness.

**Figure 2:**
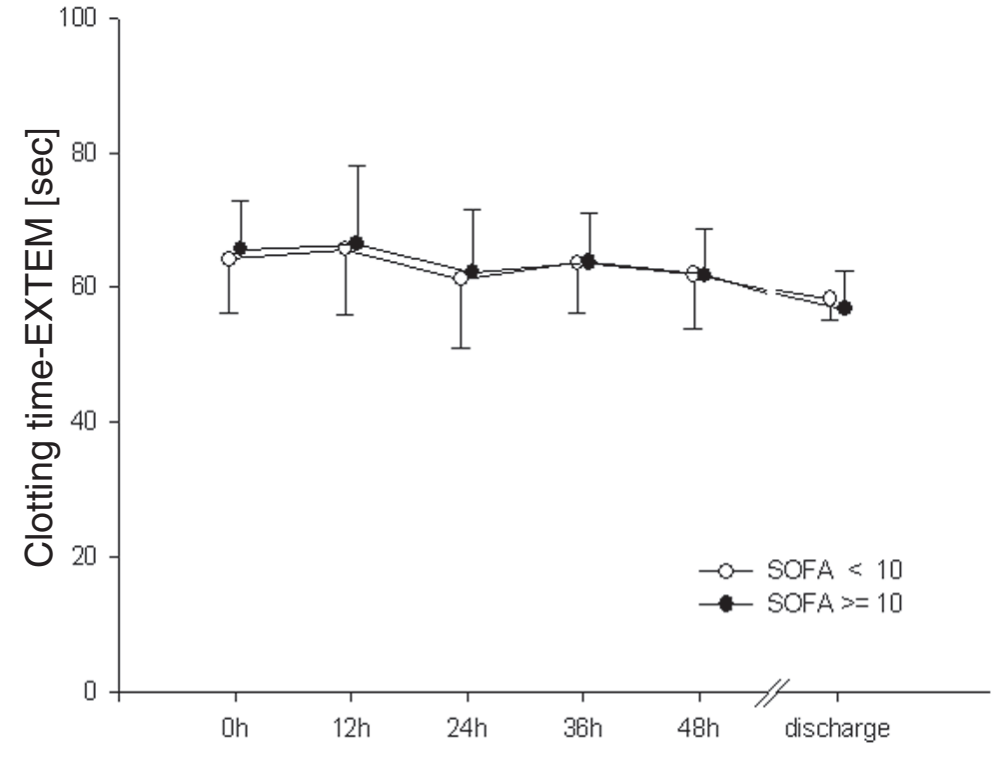
Clotting time. EXTEM, activation of coagulation with tissue factor; SOFA, Sequential Organ Failure Assessment.

**Figure 3:**
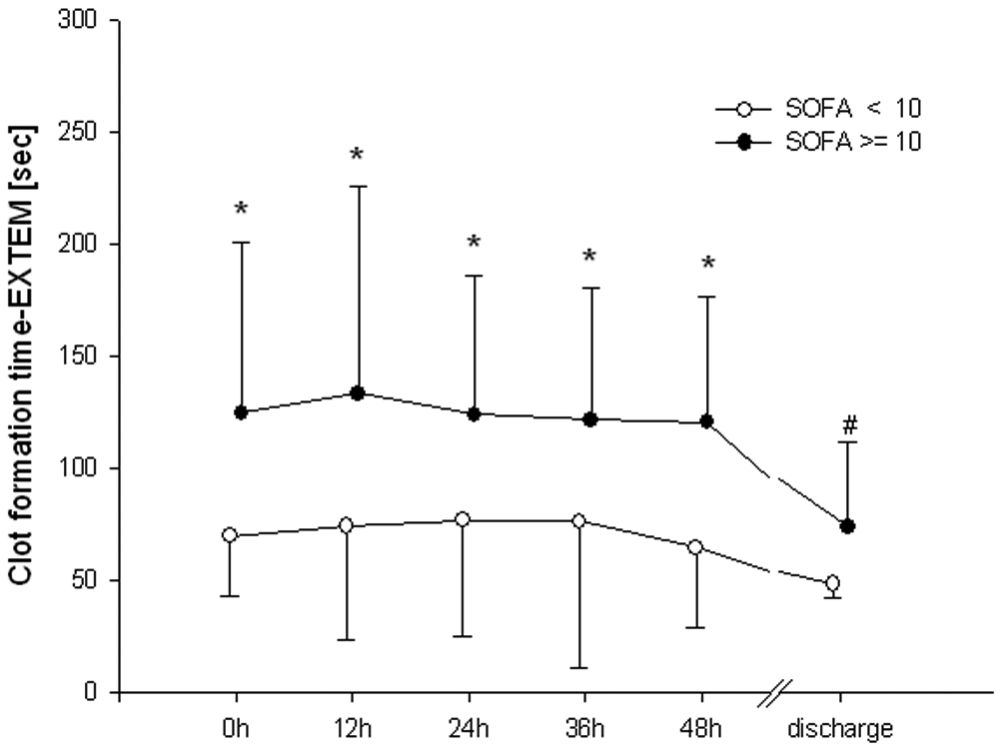
Clot formation time. *P < 0.05, differences between groups; ^#^*P *< 0.05, difference from baseline value in the high Sequential Organ Failure Assessment (SOFA; ≥ 10) group. CFT, clot formation time; EXTEM, activation of coagulation with tissue factor.

**Figure 4:**
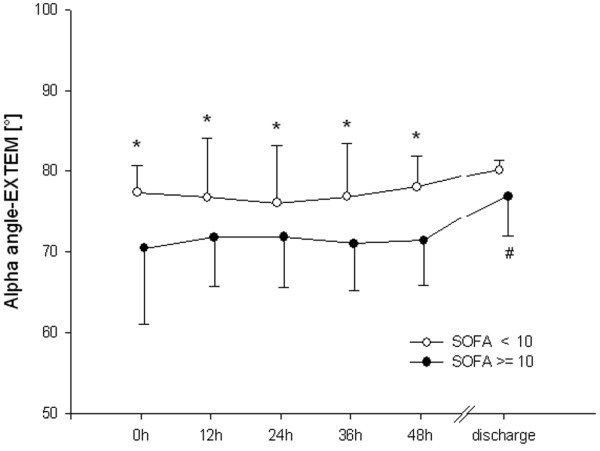
Alpha angle. *P < 0.05, differences between groups; ^#^*P *< 0.05, difference from baseline value in the high Sequential Organ Failure Assessment (SOFA; ≥ 10) group. EXTEM, activation of coagulation with tissue factor.

In patients with higher scores for organ failure, MCF differed from the low SOFA group during the first 48 hours. This difference had resolved by the time of discharge (Figure 1). CT did not differ between groups (Figure 2). Clot propagation, characterized by CFT and alpha angle, was significantly slowed in the group with the higher organ dysfunction scores compared with the low SOFA group during the first 48 hours (Figures 3 and 4). However, after 48 hours the values in the two groups converged.

**Figure 5:**
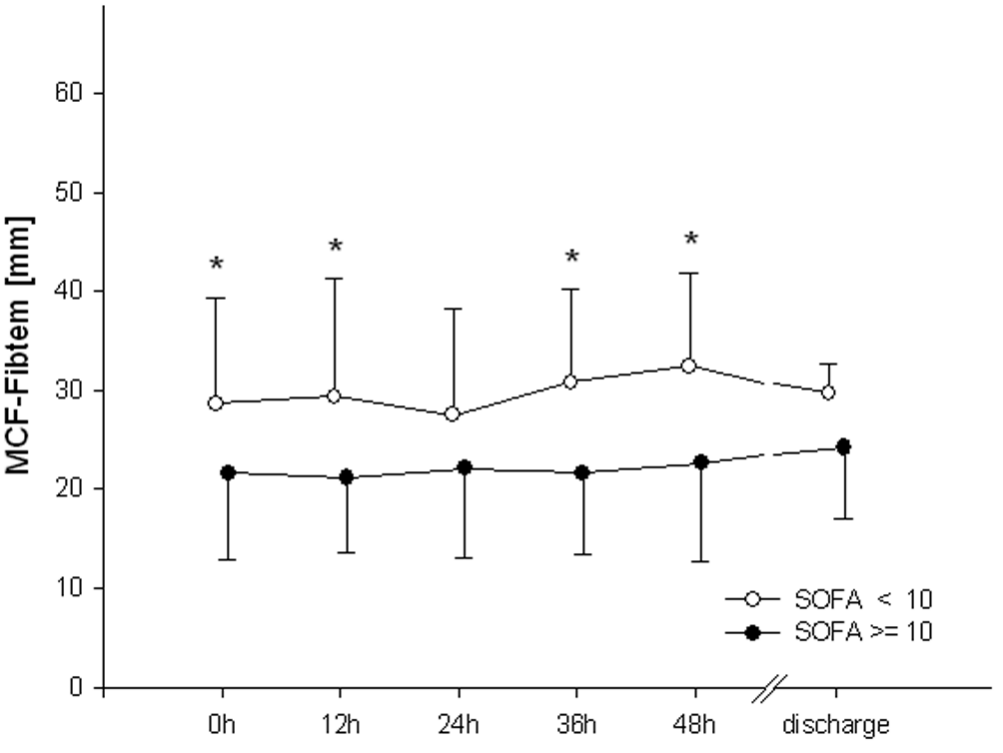
Maximal clot firmness after platelet inhibition with cytochalasin D (FIBTEM). The normal range for maximal clot firmness (MCF) is 9 to 25 mm [30]. *P < 0.05, differences between groups. SOFA, Sequential Organ Failure Assessment.

Platelet inhibition with cytochalasin revealed stronger impairment of MCF in the high SOFA group (Figure 5). The relative contribution of the fibrin clot (after inhibition of the platelet contribution with cytochalasin) remained stable during the ICU stay. No differences in the clotting time were noted between the HEPTEM and the INTEM assay results.

The variables of ROTEM^® ^thromboelastometry CFT, alpha angle, and MCF correlated moderately with disease severity defined by the SOFA score (Table 3).

## Discussion

The coagulation system is commonly activated during sepsis as a result of crossreactions with the inflammatory system [5,31]. In the present cohort study we assessed coagulation abnormalities in septic patients during the critical phase of the illness using ROTEM^® ^thromboelastometry. In order to evaluate further the relationship between perturbation of the coagulation system and organ dysfunction, we divided the cohort at the median of the initial SOFA score and compared the resulting two groups.

Improvements in SOFA scores of these patients were associated with shortened time to initiation of coagulation, accelerated clot formation, and improved firmness of the final clot at ICU discharge when compared with values on admission. Therefore, we hypothesize that the coagulation system has recovered toward the patients' individual baselines upon discharge from the ICU. Nevertheless, key thromboelastometric variables for the patients in our study remained within the range of normal reference values reported in a multicenter trial [30]. In this context, no signs of overt disseminated intravascular coagulation with adverse bleeding events were observed. Of note, compared with the increase in clot firmness, the rise in the platelet count during the course of critical illness was disproportionately high, whereas fibrinogen levels remained constant. This underlines the observation that fibrinogen levels appear to have a much greater impact on MCF than do changes in platelet count [32,33].

The results of ROTEM^® ^thromboelastometry in the present study did not suggest hypercoagulability in terms of reduced CT, reduced CFT, or increased MCF compared with established normal ranges [30,34-37]. The mean of the MCF in the FIBTEM assay of the patients with a SOFA score less than 10 was slightly higher than the reported normal range. However, because the FIBTEM assay measures only an isolated component of the overall clot firmness, it may probably not be used as a reliable parameter for hypercoagulability. In this respect, our results differ from the thromboelastographic data reported in a study by Gonano and coworkers [38], who described hypercoagulability based on their results of decreased *r *and *k *values using TEG^®^. Two other studies employing sepsis models also came to the conclusion that lipopolysaccharide-induced hypercoagulability may be detected by thromboelastography [22,23]. Spiel and coworkers [22] administered a bolus injection of endotoxin to healthy volunteers and demonstrated a transient decrease in the coagulation time, limited to the first 24 hours after injection, accompanied by increased markers of coagulation activation (increased levels of prothrombin fragments F_1+2_) [22]. Incubation of whole blood of healthy volunteers with endotoxin also produced a decrease in clotting time [23]. Clearly, the two latter investigations measured the effect of lipopolyaccharide on hemostasis in highly standardized models reflecting the very early phase after induction of inflammation. However, the clinical scenario of critically ill patients admitted to an ICU differs from these situations.

In critically ill patients with severe sepsis and septic shock, the disease has reached a more advanced stage – with advanced inflammatory response and sustained exposure to the infectious pathogen – by the time they present at the ICU. In the clinical setting, assessment of activation of coagulation by thromboelastography may thus not present with shortening of CT and CFT but rather with a development in the opposite direction, in which coagulation factors are consumed. A concomitant decrease in the production of clotting factors due to hepatic dysfunction in sepsis may also play a role, however [39].

Our data are supported by an analysis of the placebo group in the PROWESS (Recombinant Human Protein C Worldwide Evaluation in Severe sepsis) study, which demonstrated that increased severity of sepsis is accompanied by prolonged coagulation times, suggesting decreased activity of coagulation factors as a result of increased consumption [40].

Activation of coagulation is a well-known pathophysiological process in sepsis [5,14-17,31]. That accelerated clot formation and increased clot strength were not present in our study by no means excludes activation of coagulation in sepsis. The more likely explanation for our findings is that inappropriate activation of coagulation may be depicted by thromboelastography only at an early and possibly preclinical phase. However, once coagulation factors are depleted, direct thromboelastographic signs of hypercoagulation may be absent.

There is concern that the administration of colloids may have affected the coagulation system. Although the doses of fluid resuscitation with 6% hydroxethyl starch 130/0.4 differed between the two groups, the administered volumes have reportedly only minor effects on parameters of thromboelastography [41,42].

A low-dose regimen of unfractionated heparin was administered to the patients in the present study in order to prevent thromboembolic complications. This could have influenced thromboelastography analysis. However, in a heparinase assay (ROTEM^® ^HEPTEM) we analyzed clotting time as the variable of interest in this context and excluded such an effect [43]. These results suggest that the low dose of heparin used for thromboprophylaxis may not be detected by thromboelastography.

A limitation of our study is that the reported changes of ROTEM^® ^parameters had to be interpreted on the basis of external reference values. Future investigations in a controlled study may reveal clearer relationships between organ dysfunction and the coagulation system assessed by thromboelastography.

## Conclusions

Key variables for ROTEM^® ^remained within the reference ranges during the phase of critical illness in this cohort of patients with severe sepsis and septic shock without bleeding complications. Although average thromboelastometry variables did not provide additional information to standard coagulation tests, certain dynamics of ROTEM^® ^variables were noted within the reference ranges. Improved organ dysfunction upon discharge from the ICU was associated with shortened coagulation time, accelerated clot formation, and increased firmness of the formed blood clot when compared with values on admission. With increased severity of illness, changes of ROTEM^® ^variables were more pronounced.

Thromboelastography performed in patients with severe sepsis cannot reliably detect activation of coagulation in the sense of a hypercoagulable state. Further studies in patients with sepsis are warranted to investigate the role of thromboelastography in relation to bleeding and thromboembolic complications as end-points.

## Key messages

• Key thromboelastometric variables remained within reference ranges during the course of critically illness in patients with sepsis without adverse bleeding events.

• After resolution of the critical illness in patients with severe sepsis/septic shock, thromboelastometric variables exhibited shortened coagulation time, accelerated clot formation, and increased firmness of the formed blood clot when compared with values on admission. With increased severity of illness, these changes were more pronounced.

• Thromboelastometry, when performed in patients with established severe sepsis and septic shock, cannot reliably detect activation of coagulation in the sense of a hypercoagulable state, as suggested by *in vitro *or experimental studies.

## Abbreviations

aPTT: activated partial thromboplastin time; CFT: clot formation time; CT: clotting time; ICU: intensive care unit; INR: International Normalized Ratio; MCF: maximal clot firmness; SOFA: Sequential Organ Failure Assessment.

## Competing interests

The authors declare that they have no competing interests.

## Authors' contributions

FD made substantial contributions to the design of the study, analysis and interpretation of the data, and was involved in drafting the manuscript. UK, HF, and JSL made substantial contributions to the thromboelastometric measurements and to the acquisition of the data, and critically revised the manuscript for important intellectual content. JT critically revised the manuscript for important intellectual content. SMJ made substantial contributions to the concept and design of the study, and to the analysis and interpretation of the data, and was involved in drafting the manuscript. All authors read and approved the final manuscript.
